# Supplementary material for: Liquefaction of water on the surface of anisotropic two-dimensional atomic layered black phosphorus
Source: Nat Commun. 2019 Sep 6;10:4062. doi: 10.1038/s41467-019-11937-9 (PMC6731341; doi:10.1038/s41467-019-11937-9)
Supplement: Supplementary file 1 — Supplementary Information [file 41467_2019_11937_MOESM1_ESM.pdf]

Supplementary Information

Liquefaction of Water on the Surface of Anisotropic Two-dimensional Atomic Layered Black Phosphorus

Zhao *et al*.

List of Content

Supplementary Figures 1-6.

Supplementary Figures

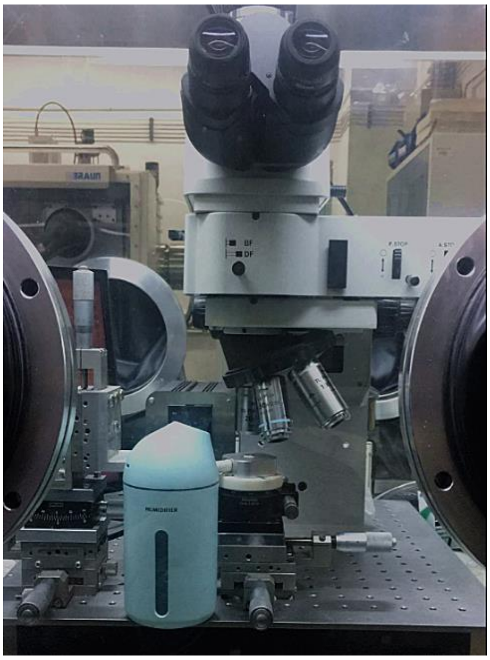

Supplementary Figure 1. The system for investigating the liquefaction process of water on the surface of BP. The system including a vapor generator and an optical microscope, which have been placed in a glove box under the protection of argon atmosphere.

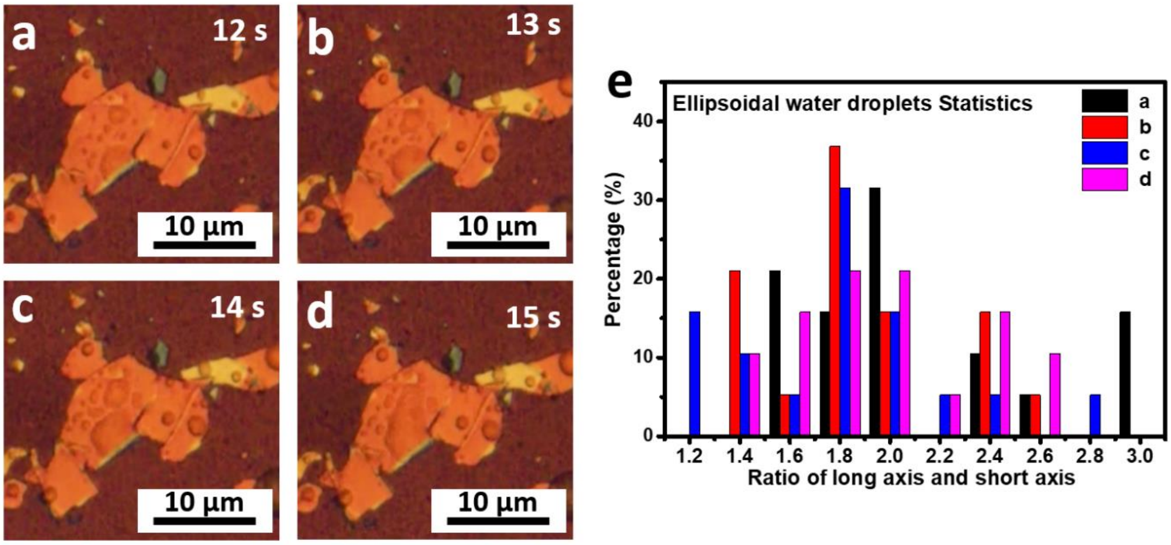

f

	a	b	c	d
Number of ellipsoidal water droplets	19	19	17	19
Number of spherical water droplets	4	4	5	6

Supplementary Figure 2. The different time screenshots in Supplementary Movie 1 and data statistics. (a) 12 s, (b) 13 s, (c) 14 s, (d) 15 s; (e) The data statistics of the long axis and short axis ratio for the elliptic water droplets on the BP layer surface in different time screenshots in Supplementary Movie 1; (f) The number of elliptic and spherical water droplets in different time screenshots in Supplementary Movie 1.

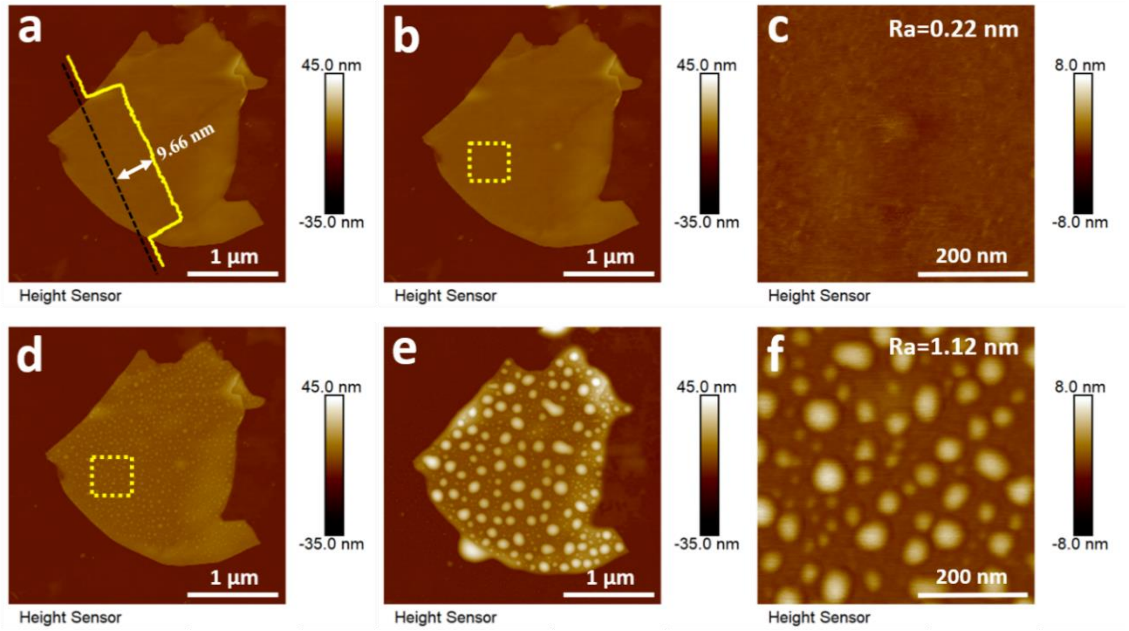

Supplementary Figure 3. Atomic force microscope images of BP nanosheets. (a) a pristine BP layer; (b) after ten times water wetting experiments; (c) a zoomed AFM image marked in the yellow dash frame in image b; (d) one day exposed in air; (e) two days exposed in air; (f) a zoomed AFM image in the yellow dash frame in image d.

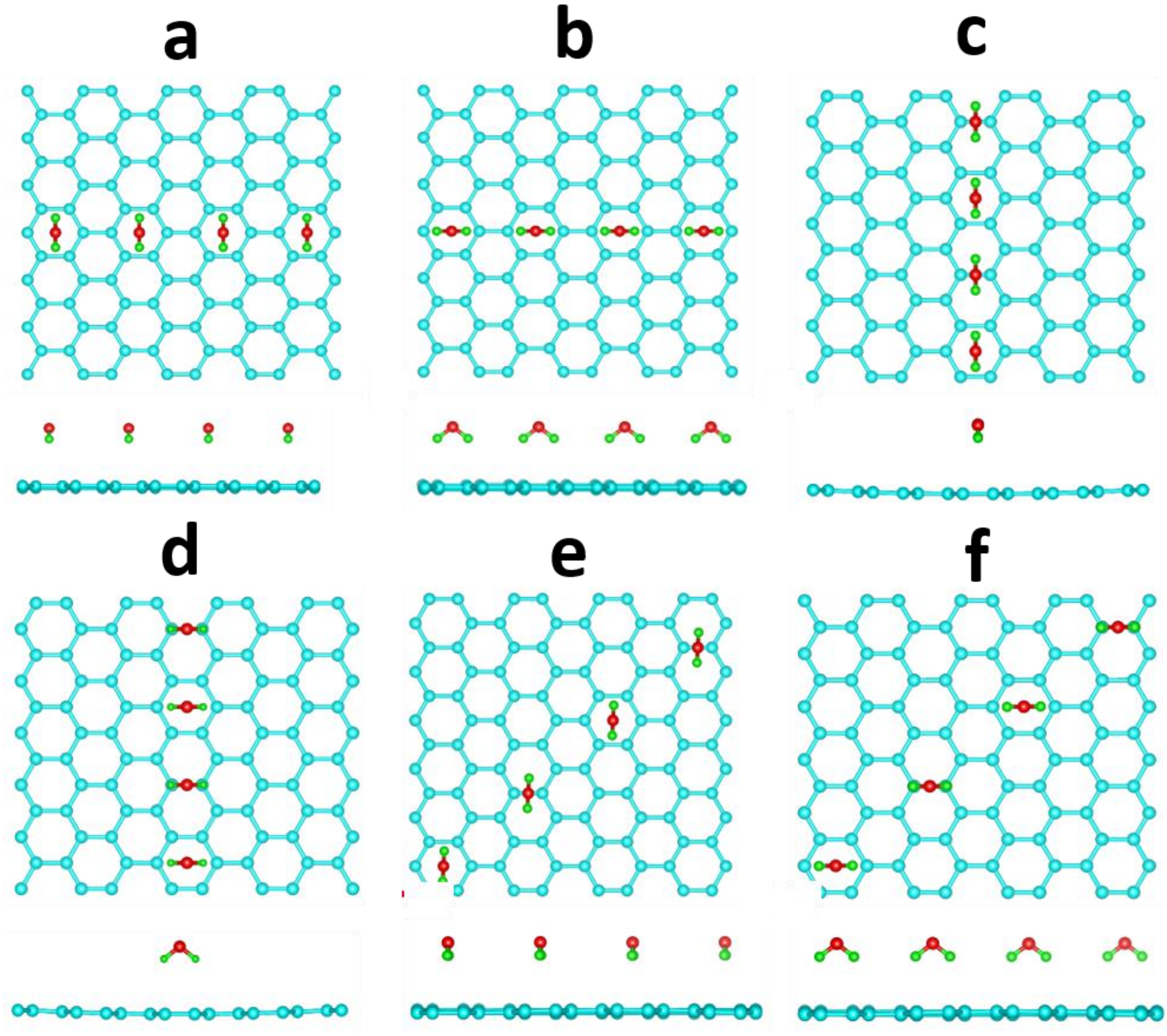

Supplementary Figure 4. Six representative high symmetry configurations for H_2O molecules on graphene. (a, b) along the armchair direction, (c, d) along the zigzag direction and (e, f) along the diagonal direction; The energy difference is (a) 0.0285 eV molecule^{-1}, (b) 0.0109 eV molecule^{-1}, (c) 0.0633 eV molecule^{-1}, (d) 0.0341 eV molecule^{-1}, (e) 0.0075 eV molecule^{-1}, (f) 0 eV molecule^{-1}, respectively.

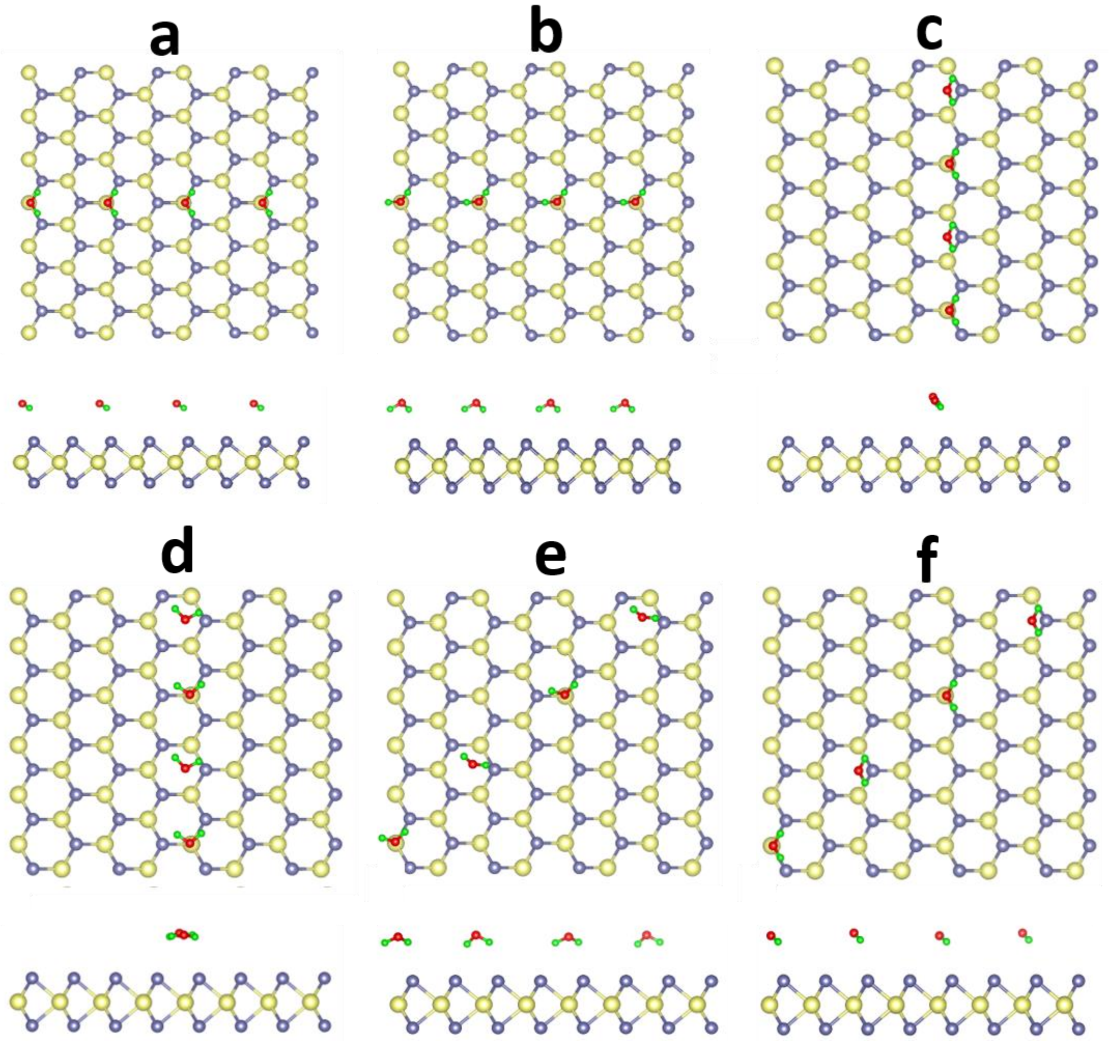

Supplementary Figure 5. **Six representative high symmetry configurations for H_2O molecules on MoS_2.** (a, b) along the armchair direction, (c, d) along the zigzag direction and (e, f) along the diagonal direction; The energy difference is (a) 0.0107 eV molecule^{-1}, (b) 0 eV molecule^{-1}, (c) 0.0513 eV molecule^{-1}, (d) 0.0032 eV molecule^{-1}, (e) 0.0083 eV molecule^{-1}, (f) 0.0373eV molecule^{-1}, respectively.

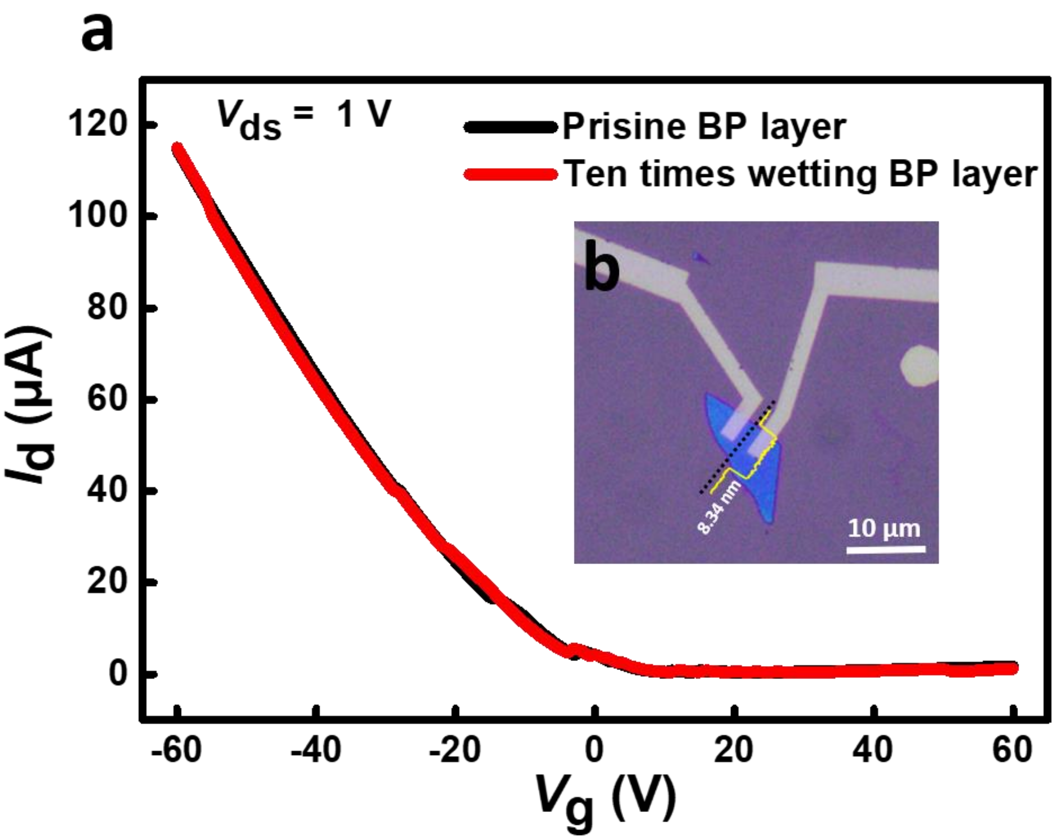

Supplementary Figure 6. The V_g and I_d curves obtained from the BP FET. (a) The V_g and I_d curves of pristine BP layer (black line) and after ten times wetting experiments BP layer (red line). (b) The optical image of the BP FET.
