# Supplementary material for: Liquefaction of water on the surface of anisotropic two-dimensional atomic layered black phosphorus
Source: Nat Commun. 2019 Sep 6;10:4062. doi: 10.1038/s41467-019-11937-9 (PMC6731341; doi:10.1038/s41467-019-11937-9)
Supplement: Supplementary file 3 — Description of Additional Supplementary Files [file 41467_2019_11937_MOESM3_ESM.docx]

**Description of Additional Supplementary Files**

**File Name: Supplementary Movie 1**

**Description:** BP nano sheets with water droplets.

**File Name: Supplementary Movie 2**

**Description:** Graphene nano sheets with water droplets

**File Name: Supplementary Movie 3**

**Description:** MoS2 nano sheets with water droplets.
